# Valores de normalidade para o teste de discriminação de dois pontos para os dedos da mão na população brasileira: Estudo transversal

**DOI:** 10.1055/s-0046-1829140

**Published:** 2026-10-23

**Authors:** Mariana Ayumi Fujisaki, Vinícius Ynoe de Moraes, Fernando Araújo Pires, João Baptista Gomes dos Santos, Flávio Faloppa

**Affiliations:** 1 Grupo de Cirurgia da Mão, Departamento de Ortopedia e Traumatologia, Escola Paulista de Medicina, Universidade Federal do Estado de São Paulo, São Paulo, SP, Brasil

**Keywords:** mãos/cirurgia, neuropatia, semiologia, hand/surgery, neuropathy, semiology

## Abstract

**Objetivo:**

Determinar valores normativos para o teste de discriminação de dois pontos na população brasileira.

**Métodos:**

Estudo transversal em população adulta sem condições neurológicas que afetam as mãos. A discriminação (polpa digital, do polegar ao dedo mínimo) foi realizada com discriminador de dois pontos metálico, graduado e com pontas fixas. Para a determinação de normalidade, associou-se o teste de Von Frey (estesiômetro de monofilamentos de náilon calibrados).

**Resultados:**

Foram avaliadas 984 mãos, sendo 244 indivíduos do sexo masculino e 248 do sexo feminino. Quanto à ocupação, foram avaliados 354 trabalhadores não braçais, 103 braçais e 35 aposentados. Todos os dedos apresentaram intervalo de discriminação entre 2 e 5 mm. Dentre eles, a moda do 1°, 4° e 5° dedos foi de 4 mm, a do 2° e 3° dedos foi de 3 mm.

**Conclusão:**

Todos os dedos apresentaram intervalo de discriminação entre 2 e 5 mm. A moda foi de 4 mm para o 1°, 4° e 5° dedos e de 3 mm para o 2° e 3° dedos.

## Abstract (en)

**Objective:**

To establish normative values for the two-point discrimination test in the Brazilian population.

**Methods:**

The present cross-sectional study was conducted in an adult population without neurological conditions affecting the hands. Discrimination was assessed on the fingertip pulp, from the thumb to the little finger, using a graded metal two-point discriminator with fixed tips. Normality was determined using the Von Frey test, with an esthesiometer consisting of calibrated nylon monofilaments.

**Results:**

A total of 984 hands, comprising 244 male and 248 female participants, were evaluated. The sample included 354 non-manual workers, 103 manual workers, and 35 retired participants. All fingers showed a discrimination range from 2 to 5 mm. The modes were 4 mm, 3 mm, 3 mm, 4 mm, and 4 mm for the 1 ^st^ , 2 ^nd^ , 3 ^rd^ , 4 ^th^ , and 5 ^th^ fingers, respectively.

**Conclusion:**

All fingers showed a minimum discrimination range from 2 to 5 mm, with a mode of 4 mm.

## Introdução

O teste de discriminação de dois pontos é definido como a menor distância necessária para discriminar dois estímulos táteis distintos, sendo amplamente utilizado para avaliar a acuidade tátil de uma determinada região cutânea e, por consequência, a função sensorial dessa área. 1 2 3

Apesar de ser um teste de fácil acesso, baixo custo e ampla aplicação clínica, vários aspectos do teste de discriminação de dois pontos carecem de padronização metodológica na literatura atual. Em particular, existem variações significativas nos protocolos de aplicação, na definição de intervalos de distância utilizados e na forma como os resultados são obtidos e interpretados, o que compromete a comparabilidade entre estudos e entre diferentes serviços de saúde. 4

Nesse contexto, Moberg 5 relatou que havia descoberto essa técnica há 45 anos e que foi um grande erro não tê-la descrito em detalhes na época. A pressão necessária para realizar o exame é um ponto de discussão importante, uma vez que é extremamente difícil padronizar a força aplicada pelo examinador para gerar o estímulo tátil suficiente sem provocar estímulo doloroso e alterar o resultado do exame. 5 6

Duas formas principais para determinar a força de aplicação são descritas: a força suficiente para que o paciente sinta o estímulo, o que está sujeito à interpretação subjetiva do participante, pois pode confundir a sensação com estímulo doloroso, e a aplicação da menor força necessária para causar alteração na coloração da pele adjacente ao contato com o instrumento (fenômeno conhecido como *blanching* ). 4 7

Para superar essa dificuldade, foi desenvolvido um dispositivo computadorizado para aplicação de pressão padronizada, que obteve bons resultados, mas possui baixa difusão nos centros clínicos. 8 Estudos também mostram que mudanças na intensidade da força aplicada podem influenciar os limiares de discriminação, indicando que a pressão do estímulo é um determinante importante na acuidade tátil medida pelo teste. 7 9

No entanto, mesmo com a força de aplicação padronizada em todas as medidas, o paciente pode ser influenciado pela alteração na percepção da pressão aplicada com um ou dois pontos, em vez da sensibilidade tátil de um ou dois pontos de contato. 10 Além disso, há falta de consenso sobre o número de vezes que cada local deve ser avaliado para um resultado mais fidedigno, variando de 3 a 10 vezes. 11 12

Os valores de referência para o teste classicamente consideram < 6 mm como normal, 13 enquanto estudos mais recentes ampliam o espectro do teste, classificando os resultados como normal (< 6 mm), razoável (6–10 mm) e inferior (11–15 mm). 14 Além disso, sexo e idade influenciam os valores de normalidade, 6 15 16 com pior desempenho conforme o envelhecimento. 6 A frequência do teste e repetições seriadas também alteram o resultado da discriminação, facilitando a percepção de dois pontos a menores distâncias, 17 mas não há estudos avaliando se houve regressão no resultado após longos períodos sem estímulos. Outros possíveis fatores de influência, como mão dominante e ocupação, não foram amplamente avaliados até o momento. Este estudo tem como objetivo determinar os valores normativos para o teste de discriminação de dois pontos na população brasileira.

## Métodos

Trata-se de um estudo transversal realizado no Grupo de Cirurgia da Mão, Departamento de Ortopedia e Traumatologia, Escola Paulista de Medicina, Universidade Federal do Estado de São Paulo. Os dados foram coletados de novembro de 2023 a maio de 2024. Este estudo foi aprovado pelo comitê de ética, sob o número CAAE: 69747623.4.0000.5505. Para a elaboração deste documento, utilizamos as diretrizes do Strengthening the Reporting of Observational Studies in Epidemiology (STROBE) (disponível em: equatornetwork.com).

Realizou-se uma análise populacional com 492 indivíduos adultos (maiores de 20 anos) saudáveis e sem doenças evidentes e/ou reportadas do escopo da cirurgia da mão, em sua maioria acompanhantes de pacientes do Serviço de Cirurgia da Mão do nosso hospital, totalizando 984 mãos.

Categorizamos a amostra por faixas etárias: 1) 20 a 40 anos, 2) 41 a 60 anos e 3) acima de 60 anos. Houve também categorização por gênero (masculino e feminino) e tipo de atividade profissional (braçais vs. não-braçais) distribuídos da seguinte forma: 1) 244 indivíduos do sexo masculino e 2) 248 do sexo feminino; dominância e ocupação, incluindo 103 trabalhadores braçais, 354 não braçais e 35 aposentados.

A fim de validar função neurológica preservada, foi realizado o teste de Von Frey com o estesiômetro de monofilamentos de náilon calibrados para avaliação da sensibilidade cutânea ( Fig. 1 ). Foram considerados como elegíveis para participação no estudo apenas os indivíduos com estesiometria verde.

**Fig. 1:**
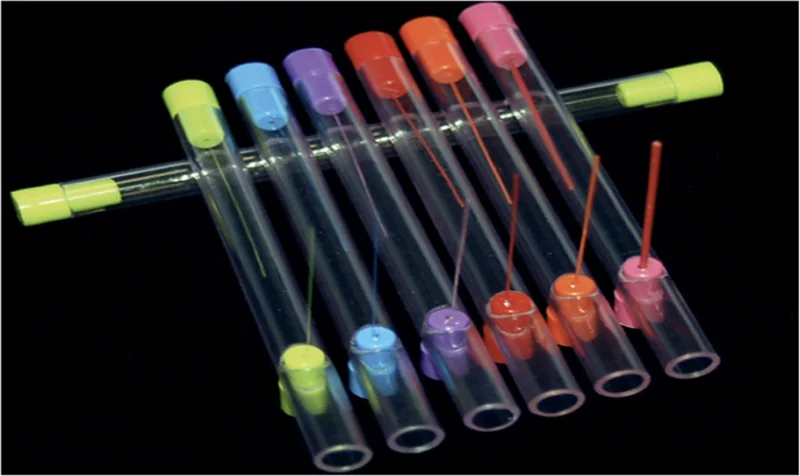
Estesiomêtro utilizado para o teste de Von Frey.

O teste foi realizado com o indivíduo sentado, punhos em posição neutra, mãos com dorso apoiado sobre a mesa e dedos em posição de repouso. Foi utilizado o discriminador de dois pontos metálico, graduado e com pontas fixas, próprio para avaliação de discriminação tátil estática ( Fig. 2 ). A medida foi realizada na polpa digital de cada dedo, do 1° ao 5°, posicionando o discriminador sobre a linha média da polpa digital longitudinalmente ( Fig. 3 ).

**Fig. 2:**
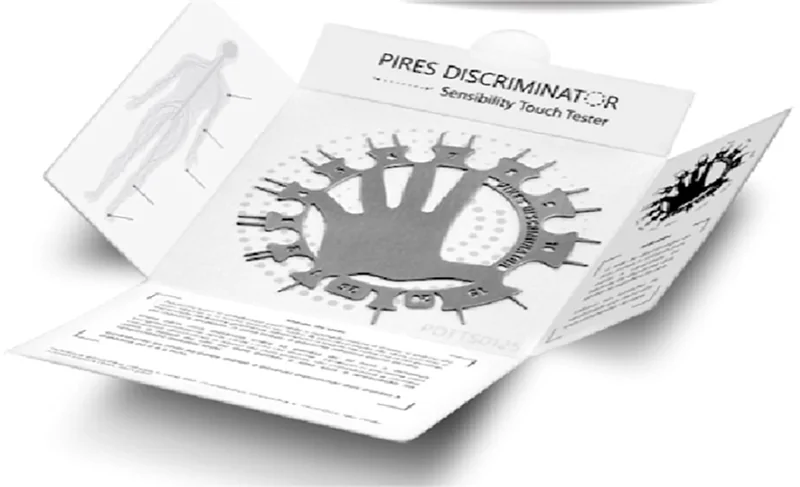
Discriminador de dois pontos metálico.

**Fig. 3:**
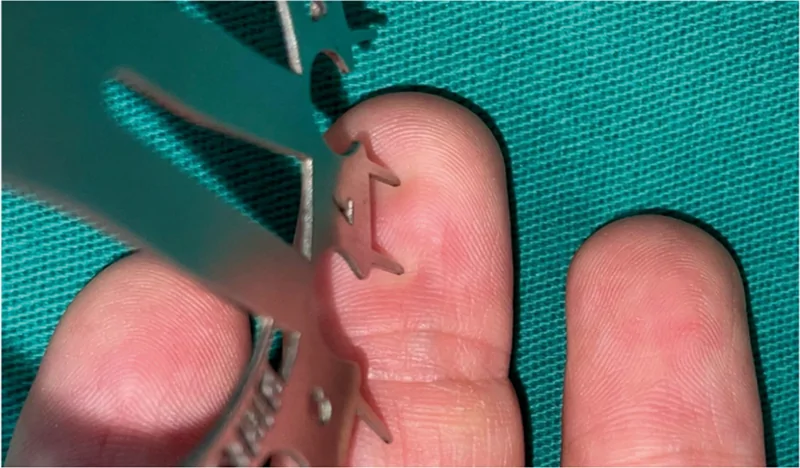
Fenômeno de blanching descrito por Moberg.

Solicitou-se ao participante do estudo que fechasse os olhos para não visualizar a realização do teste. Aleatoriamente, foi feito contato com uma ou duas pontas, de forma que tocassem a pele simultaneamente, e pediu-se ao indivíduo que indicasse se sentia toque em um ou dois pontos.

A medida inicial foi feita com 10 mm, diminuindo-se 1 mm progressivamente até que não fosse mais capaz de se discriminar dois pontos de contato. 13 A pressão do toque sobre a polpa digital foi suficiente para que a área em contato com o discriminador alterasse a coloração da pele, fenômeno descrito como *blanching* por Moberg ( Fig. 4 ). Foi considerado para registro o valor da última distância, em milímetros (mm), em que o paciente diferenciou dois pontos antes de referir apenas um ponto de contato.

**Fig. 4:**
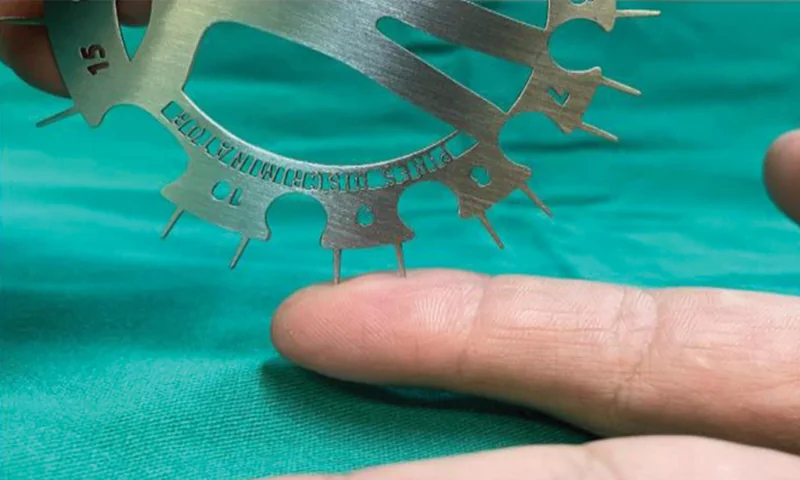
A medida foi realizada na polpa digital de cada dedo, do 1° ao 5° dedo, posicionando o discriminador sobre a linha média da polpa digital longitudinalmente.

Todos os dados foram submetidos a análise estatística para identificar correlações relevantes. Foram aplicados os testes análise de variância ( *analysis of variance* , ANOVA, em inglês) para comparar a discriminação entre os dedos na amostra total, segmentando também por faixa etária, sexo e tipo de ocupação. Para comparar os mesmos dedos entre as mãos dominante e não dominante, foi utilizado o teste *t* de Student pareado. Para realizar a comparação dos dedos aos pares, utilizou-se o método de comparação múltipla de Tukey. O nível de significância adotado foi de 5%.

## Resultados

A moda da distância entre dois pontos na população brasileira foi de 4 mm e sua distribuição dedo a dedo é demonstrada nas Figs. 5 6 .

**Fig. 5:**
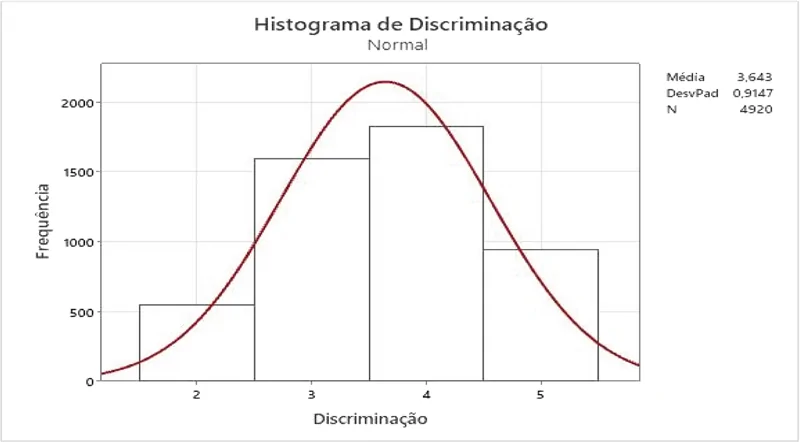
Distribuição da distância entre dois pontos dedo a dedo na população brasileira.

**Fig. 6:**
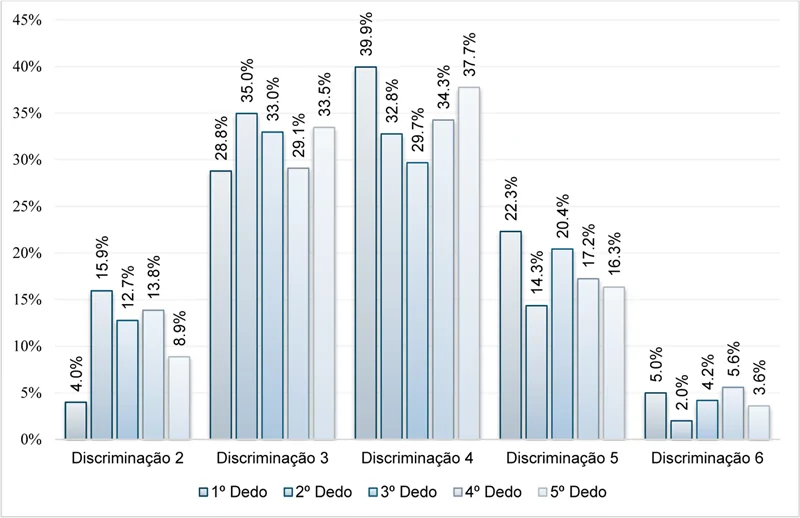
Distribuição da discriminação por dedo (todos).

As médias e seus respectivos desvios padrão, assim como a moda e intervalo de confiança, estão apresentados na Tabela 1 para a população brasileira, na Tabela 2 de acordo com a faixa etária, na Tabela 3 para os sexos masculino e feminino, na Tabela 4 conforme a ocupação dos indivíduos avaliados e, por fim, na Tabela 5 em relação à dominância das mãos.

**Tabela 1:** Discriminação entre dois pontos dedo a dedo (n = 984)

		Discriminação min–máx	Média (CI95%)	Desvio padrão	Moda	Valor de *p*
	1° dedo	2–5	3,870 (3,819–3,921)	0,821	4	
	2° dedo	2–5	3,466 (3,408–3,524)	0,931	3	
Todos	3° dedo	2–5	3,612 (3,552–3,672)	0,960	3	< 0,001
	4° dedo	2–5	3,624 (3,564–3,684)	0,957	4	
	5° dedo	2–5	3,641 (3,588–3,694)	0,850	4	

**Tabela 2:** Discriminação entre dois pontos por faixas etária

		Média (CI95%)	Desvio padrão	Moda	Valor de *p*
	1° dedo	3,650 (3,589–3,711)	0,755	4	
	2° dedo	3,176 (3,110–3,242)	0,820	3	
20–40	3° dedo	3,282 (3,213–3,351)	0,855	3	< 0,001
	4° dedo	3,370 (3,300–3,440)	0,865	3	
	5° dedo	3,432 (3,370–3,494)	0,771	3	
	1° dedo	4,097 (3,991–4,203)	0,853	4	
	2° dedo	3,794 (3,679–3,909)	0,928	4	
41–60	3° dedo	3,992 (3,877–4,107)	0,926	5	< 0,001
	4° dedo	3,883 (3,758–4,008)	1,005	4	
	5° dedo	3,863 (3,749–3,977)	0,916	4	
	1° dedo	4,382 (4,269–4,495)	0,690	5	
	2° dedo	4,097 (3,955–4,239)	0,872	5	
≥ 61	3° dedo	4,313 (4,183–4,443)	0,797	5	< 0,001
	4° dedo	4,222 (4,083–4,361)	0,848	5	
	5° dedo	4,118 (3,995–4,241)	0,753	4	

**Tabela 3:** Discriminação entre dois pontos por sexo

		Média (CI95%)	Desvio padrão	Moda	Valor de *p*
	1° dedo	3,911 (3,836–3,986)	0,855	4	
	2° dedo	3,522 (3,441–3,603)	0,921	3	
Feminino	3° dedo	3,677 (3,596–3,758)	0,917	4	< 0,001
	4° dedo	3,679 (3,596–3,762)	0,943	4	
	5° dedo	3,641 (3,564–3,718)	0,876	4	
	1° dedo	3,828 (3,758–3,898)	0,784	4	
	2° dedo	3,410 (3,327–3,493)	0,940	3	
Masculino	3° dedo	3,545 (3,456–3,634)	0,998	3	< 0,001
	4° dedo	3,568 (3,482–3,654)	0,970	4	
	5° dedo	3,641 (3,568–3,714)	0,823	4	

**Tabela 4:** Discriminação entre dois pontos por ocupação

		Média (CI95%)	Desvio padrão	Moda	Valor de *p*
	1° dedo	4,248 (4,154–4,342)	0,686	4	
	2° dedo	3,990 (3,868–4,112)	0,894	4	
Trabalhador braçal	3° dedo	4,121 (4,002–4,240)	0,872	5	< 0,001
	4° dedo	4,117 (4,005–4,229)	0,818	4	
	5° dedo	4,015 (3,900–4,130)	0,841	4	
	1° dedo	3,701 (3,642–3,760)	0,802	4	
	2° dedo	3,237 (3,175–3,299)	0,843	3	
Trabalhador não braçal	3° dedo	3,363 (3,298–3,428)	0,887	3	< 0,001
	4° dedo	3,400 (3,333–3,467)	0,915	3	

**Tabela 5:** Discriminação entre dois pontos por dominância

		Média (CI95%)	Desvio padrão	Moda	Valor de *p*
	Dominante	3,868 (3,791–3,945)	0,873	4	
1° dedo					0,914
	Não dominante	3,872 (3,804–3,940)	0,767	4	
	Dominante	3,437 (3,353–3,521)	0,954	3	
2° dedo					0,124
	Não dominante	3,496 (3,416–3,576)	0,908	3	
	Dominante	3,591 (3,508–3,674)	0,943	3	
3° dedo					0,304
	Não dominante	3,632 (3,546–3,718)	0,977	4	
	Dominante	3,606 (3,520–3,692)	0,976	4	
4° dedo					0,410
	Não dominante	3,642 (3,559–3,725)	0,939	4	
	Dominante	3,602 (3,527–3,677)	0,849	4	
5° dedo					0,021
	Não dominante	3,681 (3,606–3,756)	0,850	4	

Todos os dedos apresentaram intervalo de discriminação entre 2 e 5 mm. Dentre eles, a moda do 1°, 4° e 5° dedos foi 4 mm, a do 2° e 3° dedos.

Dentre todos os dedos avaliados, o segundo dedo apresentou menores distâncias de discriminação em comparação aos demais.

Em relação à faixa etária, observou-se variação conforme a idade ( Tabela 2 ). Os mais jovens (20–40 anos), totalizando 296 indivíduos, apresentaram menores distâncias de discriminação em comparação aos 124 com idades entre 41 e 60 anos e aos 72 maiores de 60 anos.

Os 354 trabalhadores não braçais exibiram melhores capacidades de discriminação em comparação aos 103 trabalhadores braçais, sendo os aposentados, 35 no total, os que apresentaram a menor capacidade ( Tabela 4 ). Não houve diferença significativa na capacidade de discriminação entre os 244 homens e 248 mulheres avaliados, e entre mãos dominantes e não dominantes houve apenas no 5° dedo. ( Tabelas 3 5 ).

## Discussão

Considerando a escassez de padronização na população brasileira, este estudo avaliou 984 mãos não neuropáticas e demonstrou valores entre 2 e 5 mm, corroborando achados prévios, 18 com moda de 4 mm no 1°, 4° e 5° dedos, e 3 mm no 2° e 3° dedos. Para assegurar função neurológica preservada, utilizou-se o teste do monofilamento de von Frey, incluindo apenas indivíduos com estesiometria verde. 19 20

Apesar dos critérios clínicos de exclusão, reconhecem-se limitações do monofilamento, cuja sensibilidade para neuropatias iniciais é variável, podendo não detectar alterações subclínicas. 21 Ademais, há variação descrita em sua acurácia diagnóstica, com possibilidade de falsos negativos e falsos positivos quando utilizado como método isolado de triagem. 22 Assim, não se pode excluir totalmente a inclusão de alterações sensoriais leves não detectadas.

O segundo dedo apresentou menores distâncias de discriminação, indicando maior sensibilidade tátil, possivelmente relacionada à maior densidade de receptores nas regiões distais e mais utilizadas funcionalmente. 23 Quanto mais ricamente inervado é o dedo, mais clara e corretamente será possível sentir a diferença entre dois pontos tocados. 18 23

A correlação observada entre o aumento da idade e o pior desempenho sensorial corrobora achados prévios da literatura, que descrevem um declínio progressivo da sensibilidade com o envelhecimento. 16 Evidencia-se que a capacidade de discriminação de dois pontos tende a deteriorar-se ao longo dos anos, fenômeno associado à redução quantitativa e funcional dos receptores cutâneos, especialmente dos corpúsculos de Meissner e das células de Merkel. 16 Estudos histológicos clássicos conduzidos por Cauna e Ross 24 e Ridley 25 demonstram essa diminuição relacionada à idade, oferecendo embasamento morfofuncional para a observação de limiares mais elevados na discriminação de dois pontos em indivíduos mais velhos.

Não houve diferença significativa entre os sexos, em consonância com parte da literatura. Contudo, um estudo norte-americano identificou que as mulheres obtiveram pontuação inferior à dos homens ( *p* < 0,001). 26 Os autores observaram maiores limiares em mulheres, além de melhor desempenho nas pontas dos dedos. 26 A ausência de relevância estatística em relação a dominância das mãos corrobora os achados de Cheng. 14 Dessa maneira, sugere-se que a dominância não exerce um impacto significativo na discriminação de dois pontos.

Além disso, variações metodológicas entre estudos como força aplicada, orientação do instrumento, critérios de resposta e treinamento do examinador, contribuem para divergências nos resultados descritos na literatura. A padronização metodológica adotada neste estudo, incluindo o uso prévio do teste do monofilamento de Von Frey para exclusão de neuropatias subclínicas, permitiu definir valores mais consistentes e específicos para a população brasileira, situados entre 2 e 5 mm, refinando e corroborando a literatura clássica.

Como limitação, destaca-se o delineamento transversal, que impede análises causais ou longitudinais, e a escassez de estudos recentes sobre a padronização do teste em populações saudáveis. Ainda assim, os fundamentos fisiológicos e metodológicos clássicos permanecem válidos. Estudos futuros, com amostras mais diversas e metodologias uniformes, poderão ampliar e atualizar os parâmetros normativos propostos.

## Conclusão

A discriminação de 2 pontos na população brasileira em mãos saudáveis variou de 2 a 5 mm e a moda de 4 mm. A moda foi de 4 mm para o 1°, 4° e 5° dedos e 3 mm para o 2° e 3° dedos. O segundo dedo apresentou menores distâncias de discriminação em comparação aos outros dedos. Os indivíduos mais jovens, na faixa etária de 20 a 40 anos, demonstraram menores distâncias de discriminação quando comparados aos grupos etários mais velhos. Os trabalhadores não braçais exibiram melhores capacidades de discriminação de dois pontos em comparação aos trabalhadores braçais, seguidos pelos aposentados.
